# The Association Between Corrective Surgery for Adult Spinal Deformity and Serum Levels of Hepatobiliary Enzymes

**DOI:** 10.7759/cureus.43444

**Published:** 2023-08-14

**Authors:** Kotaro Sakashita, Toshiaki Kotani, Tsuyoshi Sakuma, Yasushi Iijima, Kohei Okuyama, Shuhei Ohyama, Shohei Minami, Seiji Ohtori, Masao Koda, Masashi Yamazaki

**Affiliations:** 1 Department of Orthopedic Surgery, Institute of Medicine, University of Tsukuba, Tsukuba, JPN; 2 Department of Orthopedic Surgery, Seirei Sakura Citizen Hospital, Sakura, JPN; 3 Department of Orthopedic Surgery, Graduate School of Medicine, Chiba University, Chiba, JPN

**Keywords:** kyphotic deformity, spine deformity surgery, acute celiac artery syndrome, complications, adult spinal deformity

## Abstract

Background and objective

The surgery to correct adult spinal deformity (ASD) is associated with a rare, but life-threatening complication called acute celiac artery compression syndrome (ACACS). To our knowledge, there is currently no study in the literature regarding the abnormal elevation of serum levels of hepatobiliary enzymes after surgery to correct the deformity. In light of this, the purpose of this study was to investigate this potential association.

Materials and methods

We collected data on 74 patients with ASD who underwent correction surgery at our institution. A Spearman's rank-order correlation was used to assess the association between serum levels of hepatobiliary enzymes and spinal parameters. Factors showing a correlation coefficient of 0.2 or more were combined in a stepwise multiple regression analysis.

Results

The mean age of the patients was 68.4 ± 7.7 years; the study comprised six men and 68 women. In our stepwise multiple regression analysis, there were two valid models that included spinal parameters as independent variables: changes in lactate dehydrogenase (LDH) - changes in thoracolumbar kyphosis (TLK) (B -0.0025 ± 0.0007, p<0.01), and changes in LDH - preoperative T12-L1 kyphosis (B 0.0031 ± 0.001, p<0.01). Additionally, both valid models contained median arcuate ligament (MAL) overlap defined as MAL crossing the base of the celiac artery (CA) as a significant independent variable.

Conclusions

Greater sagittal correction of TLK, larger preoperative T12-L1 kyphosis, and MAL overlap were factors associated with an elevated serum level of LDH. Although few patients were reported to have clinically severe symptoms, “potential” ACACS due to a temporary blood flow disturbance can occur in this patient population.

## Introduction

Corrective surgery for adult spinal deformity (ASD) has benefitted from the introduction of new instruments, such as pedicle screws, to improve deformity correction. Lateral lumbar interbody fusion (LLIF) is a minimally invasive and convenient procedure and helps to accomplish a better correction than other methods. However, LLIF is associated with various complications, and their details are not yet widely known [1-3].

A rare, but life-threatening complication of surgery to correct the spinal deformity is acute celiac artery compression syndrome (ACACS) [4]. The correction of thoracolumbar kyphosis (TLK) can cause this syndrome. Aside from ACACS, postoperative ileus (POI) and an elevation of the serum level of amylase can occur after spinal surgery [5,6]. These transient abdominal organ failures have also been reported to be associated with TLK correction. The failures may be attributed to a mechanism similar to ACACS, although the detailed pathogenesis is unclear.

We speculated that if TLK correction causes celiac artery (CA) compression, an elevation of hepatobiliary enzymes would occur after the surgery to correct spinal deformity. A previous case report has recommended that if there is an abnormal elevation of serum levels of hepatobiliary enzymes after surgery to correct spinal deformity, detailed investigations should be performed to diagnose ACACS [7]. However, to our knowledge, there is currently no study of serum levels of hepatobiliary enzymes after deformity correction surgery and their association with spinal alignment. Therefore, this study aimed to investigate serum levels of hepatobiliary enzymes and spinal parameters after surgery to correct ASD.

## Materials and methods

Approval for this study was obtained from our institutional ethics review board and we adhered to the principles of the Declaration of Helsinki in conducting this study. We obtained informed consent from all patients for publication.

We retrospectively collected data on patients with ASD who had undergone corrective surgery at our institution from December 2013 to February 2020. The ASD was defined based on radiographic spinal parameters according to the SRS-Schwab ASD classification: pelvic incidence minus lumbar lordosis (PI-LL) >10°, sagittal vertical axis (SVA) >4 cm, pelvic tilt (PT) >20°, and/or coronal Cobb angle >30°. The inclusion criteria were as follows: (1) combined anterior and posterior surgery, (2) distal fixation to the pelvis, and (3) proximal fixation to L2 or higher (at least four fused segments). The exclusion criteria were as follows: revision or additional surgery; patients with an abdominal condition that would affect the elevation of hepatobiliary enzymes; and patients with a history of abdominal surgery. We analyzed data from 74 patients who satisfied the criteria. The serum levels of hepatobiliary enzymes, including aspartate aminotransferase (AST), alanine aminotransferase (ALT), alanine phosphatase (ALP), lactate dehydrogenase (LDH), and γ-glutamyl transferase (γGTP), had been measured before and one week after surgery.

The radiographic parameters as defined by the hybrid Scoliosis Research Society-Schwab classification were chosen [8]. This classification uses anterior-posterior and lateral standing radiographs of the whole spine. The same skilled spine surgeon reviewed all radiographs. The anterior-posterior view was used to measure the degree of coronal Cobb angle of the main curve. The following parameters were measured from the lateral view: SVA, thoracic kyphosis (TK: T5-T12 angle), TLK (T10-L2 angle), T12-L1 kyphosis angle, and lumbar lordosis (LL: L1-S1 angle). The changes in the coronal Cobb angle of the main curve, TLK, and T12-L1 kyphosis angle were obtained by calculating the difference between the preoperative and postoperative values.

CT of the spine was obtained preoperatively in the axial plane with 1.25 mm slice thickness, and two-dimensional reconstruction with the Synapse Vincent system (Fujifilm Medical, Tokyo, Japan). The median arcuate ligament (MAL) was evaluated with CT images. The MAL is an arch-like fascial structure of the diaphragm linking the right and left diaphragmatic crura [9]. When MAL was inferior to the base of the celiac trunk, we defined the condition as MAL overlap, based on a previous study [10].

Serum levels of hepatobiliary enzymes were distributed asymmetrically. Therefore, these data were transformed logarithmically to base 10. Changes in serum levels of hepatobiliary enzymes were obtained by calculating the differences between the preoperative and one-week postoperative values. A Spearman's rank-order correlation was used to assess the association between changes in serum levels of hepatobiliary enzymes and spinal parameters. Factors with a correlation coefficient of 0.2 or more were then combined in a multiple regression analysis. A stepwise multiple regression analysis was conducted. The dependent variable was the changes in hepatobiliary enzymes. Independent variables were age, sex, liver disease, total estimated blood loss, log-preoperative serum value of hepatobiliary enzyme (corresponding to dependent variables), MAL overlap, and each spinal parameter.

All statistical analyses were performed using EZR version 1.55 (Saitama Medical Center, Jichi Medical University, Saitama, Japan), a graphical user interface for R (The R Foundation for Statistical Computing, Vienna, Austria, version 3.6.2). More precisely, it is a modified version of the R commander software (version 2.6-1) designed to add statistical functions frequently used in biostatistics. A p-value <0.05 was considered statistically significant [11].

## Results

The mean age of the patients in the present study was 68.4 ± 7.7 years, and the cohort comprised six men and 68 women. Table 1 shows the demographic data of the patients, their spinal parameters, and serum levels of hepatobiliary enzymes.

**Table 1:** Demographic data of the patients Values are expressed as the number of patients (%) or means ± standard deviation UIV: upper instrumented vertebra; SVA: sagittal vertical axis; TK: thoracic kyphosis; TLK: thoracolumbar kyphosis; LL: lumbar lordosis; AST: aspartate aminotransferase; ALT: alanine aminotransferase; ALP: alkaline phosphatase; LDH: lactate dehydrogenase; γGTP: γ-glutamyl transpeptidase; MAL: median arcuate ligament

Variables (n=74)		Values
Age, years		68.4 (7.7)
Sex	Male	6 (8.1)
	Female	68 (91.9)
Liver disease	-	70 (94.6)
	+	4 (5.4)
UIV	T2	3 (4.1)
	T3	8 (10.8)
	T4	5 (6.8)
	T5	7 (9.5)
	T8	1 (1.4)
	T9	22 (29.7)
	T10	20 (27.0)
	L1	1 (1.4)
	L2	7 (9.5)
Number of posterior fused segments	5	7 (9.5)
	6	1 (1.4)
	9	20 (27.0)
	10	22 (29.7)
	11	1 (1.4)
	14	7 (9.5)
	15	5 (6.8)
	16	8 (10.8)
	17	3 (4.1)
Number of anterior fused segments	2	1 (1.4)
	3	8 (10.8)
	4	31 (41.9)
	5	27 (36.5)
	6	7 (9.5)
Two-staged surgery	-	6 (8.1)
	+	68 (91.9)
Total operative time, minutes		598.6 (142.0)
Total estimated blood loss, mL		897.1 (646.1)
Preoperative	AST	22.0 (6.9)
	ALT	16.8 (8.0)
	ALP	237.3 (84.5)
	LDH	206.8 (41.0)
	γGTP	24.0 (25.8)
ΔAST		11.6 (22.0)
ΔALT		32.4 (48.1)
ΔALP		139.3 (288.9)
ΔLDH		116.9 (72.6)
ΔγGTP		38.7 (60.1)
Preoperative	Cobb	38.7 (23.0)
	SVA	12.5 (7.8)
	TK	18.4 (15.8)
	TLK	20.3 (19.4)
	T12L1 kyphosis	10.3 (10.8)
	LL	7.0 (21.1)
Postoperative	Cobb	12.7 (9.7)
	SVA	2.9 (3.2)
	TK	33.7 (16.8)
	TLK	6.6 (10.7)
	T12L1 kyphosis	4.9 (5.4)
	LL	40.0 (10.2)
MAL overlap	-	40 (55.6)
	+	32 (44.4)

Table 2 shows Spearman's rank-order correlation coefficients, which define the association between changes in serum levels of hepatobiliary enzymes and spinal parameters.

**Table 2:** Spearman's rank-order correlation coefficients between changes in serum levels of hepatobiliary enzymes and spinal parameters Correlations were assessed using Spearman's rank-order correlation coefficient (rs) *rs >0.2 SVA: sagittal vertical axis; TK: thoracic kyphosis; TLK: thoracolumbar kyphosis; LL: lumbar lordosis; AST: aspartate aminotransferase; ALT: alanine aminotransferase; ALP: alkaline phosphatase; LDH: lactate dehydrogenase; γGTP: γ-glutamyl transpeptidase

	⊿AST		⊿ALT		⊿ALP		⊿LDH		⊿γGTP	
	rs	P	rs	P	rs	P	rs	P	rs	P
Preoperative										
Cobb	–0.12	0.29	–0.057	0.63	–0.0081	0.95	0.37*	0.0035	0.34*	0.038
SVA	0.18	0.12	0.24*	0.036	0.16	0.18	0.018	0.88	0.4*	0.013
TK	0.11	0.35	0.076	0.52	–0.033	0.78	0.086	0.47	0.2	0.23
TLK	0.12	0.32	0.023	0.84	0.13	0.26	0.23*	0.054	0.44*	0.0056
T12L1 kyphosis	0.134	0.26	0.032	0.8	0.071	0.55	0.21*	0.079	0.36*	0.029
LL	0.054	0.65	0.055	0.64	0.031	0.8	0.015	0.9	0.14	0.4
Postoperative										
Cobb	–0.25*	0.038	–0.19	0.11	–0.11	0.36	0.16	0.18	–0.042	0.8
SVA	–0.068	0.57	–0.04	0.74	0.004	0.973	0.00093	0.99	0.0025	0.99
TK	0.012	0.92	0.024	0.84	–0.2*	0.093	0.042	0.72	0.22	0.18
TLK	0.014	0.91	–0.0055	0.96	0.061	0.607	–0.0935	0.43	0.11	0.52
T12L1 kyphosis	0.061	0.61	0.015	0.9	0.052	0.66	0.17	0.15	0.12	0.48
LL	–0.11	0.36	–0.045	0.7	–0.12	0.31	0.16	0.18	0.14	0.4
⊿Cobb	0.031	0.79	0.019	0.87	–0.028	0.82	–0.32*	0.0059	–0.38*	0.019
⊿TLK	–0.14	0.23	–0.039	0.75	-0.14	0.22	-0.34*	0.0033	-0.43*	0.0077
⊿T12L1 kyphosis	–0.12	0.3	–0.032	0.79	–0.028	0.82	–0.16	0.19	–0.32*	0.052

To consider the impact of age, sex, hepatic diseases, and total estimated blood loss on changes in hepatobiliary enzymes, a stepwise multiple regression analysis was performed [12]. Factors with a correlation coefficient of 0.2 or more were then combined in a multiple regression analysis. Table 3 shows the results of stepwise multiple regression analysis in which the models included spinal parameters as independent variables. The two models that satisfied the conditions were as follows: changes in LDH - changes in TLK (B -0.0025 ± 0.0007, p<0.01) and changes in LDH - preoperative T12-L1 kyphosis (B 0.0031 ± 0.001, p<0.01). Additionally, these two final models contained MAL overlap as a significant independent variable. In model one, the variance inflation factor (VIF) of age, sex, hepatic disease, total estimated blood loss, MAL overlap, and changes in TLK were 1.05, 1.05, 1.03, 1.03, 1.10, and 1.08, respectively. In model two, the VIF of those and preoperative T12-L1 kyphosis were 1.04, 1.06, 1.03, 1.03, 1.14, and 1.09, in the same way. There was no multicollinearity in these models.

**Table 3:** Stepwise multiple regression analysis TLK: thoracolumbar kyphosis; MAL: median arcuate ligament; LDH: lactate dehydrogenase

Analysis				
Model 1				
	B	Std. error	t	P
⊿TLK	-0.0025	0.0007	-3.7	<0.01
Total blood loss	0.000067	0.000015	4.3	<0.01
MAL overlap	0.049	0.023	2.1	0.038
Preoperative LDH	-0.52	0.14	-3.9	<0.01
Model Summary				
Multiple R square	0.31	Adjusted R square		0.24
Model 2				
	B	Std. error	t	P
Preoperative T12-L1 kyphosis	0.0031	0.0011	2.8	<0.01
MAL overlap	0.051	0.024	2.1	0.04
Preoperative LDH	-0.49	0.14	-3.5	<0.01
Model summary				
Multiple R square	0.25	Adjusted R square		0.17

## Discussion

We found that the changes in hepatobiliary enzymes after corrective surgery for ASD, especially changes in LDH, were associated with changes in TLK, preoperative T12-L1 kyphosis, and MAL overlap. If patients have MAL overlap, the greater the sagittal correction in TLK and the larger the preoperative T12-L1 kyphosis, the greater the changes in their LDH after correction surgery.

**Figure 1:**
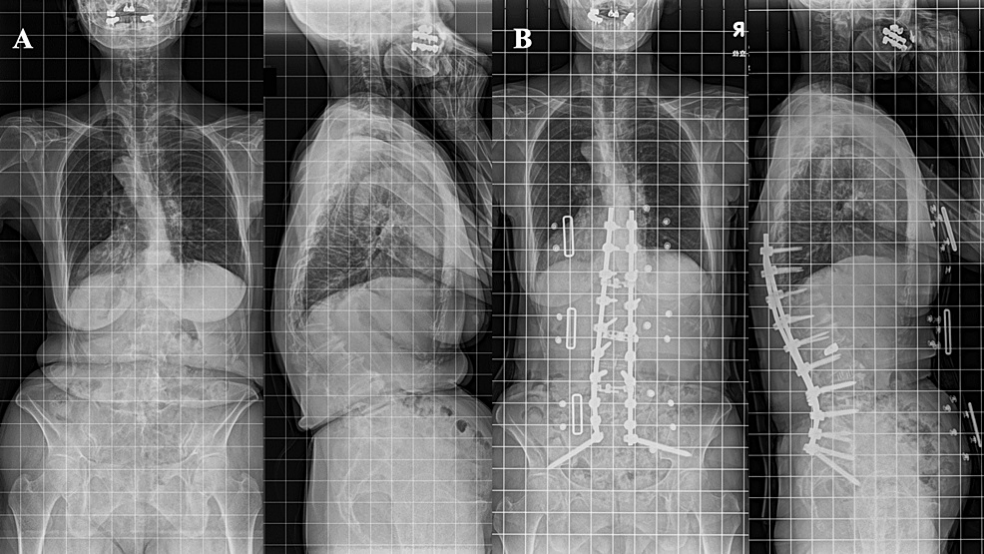
Pre- and postoperative standing-position posterior to anterior and lateral spine radiographs Preoperative (A) and postoperative (B) radiographs. A 66-year-old woman underwent combined anterior and posterior corrective surgery. The serum levels of AST, ALT, and LDH one week after surgery remained elevated, at 339, 371, and 1208 U/L, respectively AST: aspartate aminotransferase; ALT: alanine aminotransferase; LDH: lactate dehydrogenase

Two studies have demonstrated that thoracolumbar kyphosis correction surgery is associated with abdominal organ dysfunction. In the first one, of patients undergoing spinal surgery, 46% had hyperamylasemia, and its occurrence was associated with postoperative TLK and postoperative TLK changes [5]. In the second study, postoperative changes in TLK were an independent factor for POI in surgery to correct ASD [6]. In the present study, we hypothesized that specific indicators of liver dysfunction, AST or ALT, may have a relationship with correction surgery. Although we failed to establish any apparent relationship, we found that preoperative T12-L1 kyphosis and correction of TLK were associated with changes in LDH, an enzyme indicating liver function. An example is presented in Figure 1. The findings of the two previous studies [5,6] and our present study suggest that kyphosis correction surgery for thoracolumbar spinal alignment could negatively impact multiple abdominal organs. However, it was unclear why thoracolumbar kyphosis correction surgery causes abdominal organ damage. A possible mechanism is MAL overlap, which was contained in the final multiple regression analysis models. A previous study has established MAL overlap as a predisposing factor for asymptomatic stenosis of the CA before surgery [10], and hence the elevation of LDH levels may be a result of decreased liver perfusion due to asymptomatic CA stenosis.

**Figure 2:**
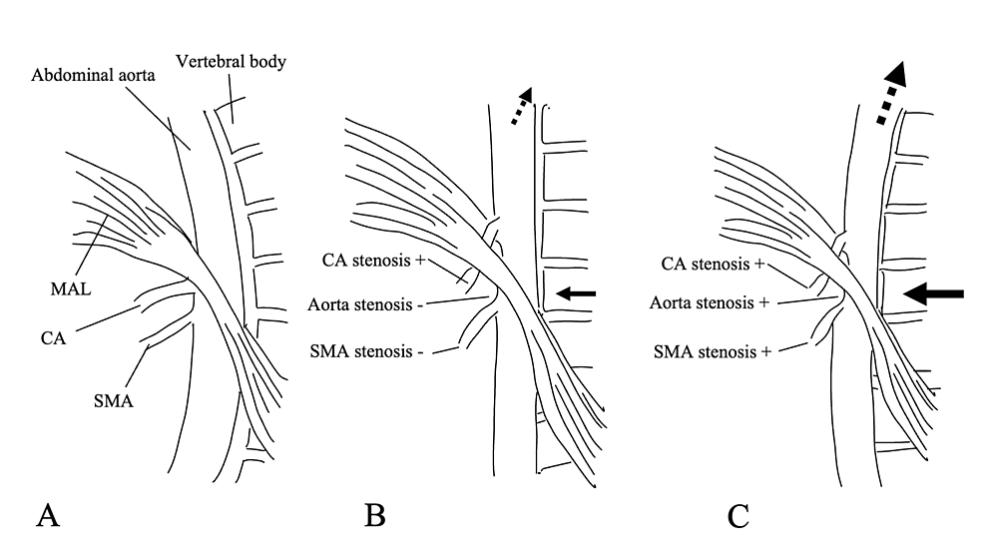
Illustration of the “conventional” and “potential” acute celiac artery compression syndrome Different mechanisms of acute celiac artery compression syndrome (ACACS) development with correction amount in the thoracolumbar spine. (A) Preoperative, (B) “potential” ACACS, and (C) “conventional” ACACS MAL: median arcuate ligament; CA: celiac artery; SMA: superior mesenteric artery

Some case reports have shown ACACS after deformity corrective surgery, resulting in multiple organ ischemia [4,7,13-17]. The development of ACACS was assumed to produce a substantial correction force, which shifted the origin of the CA upward and caused the vertebra to push the aorta forward, resulting in the CA being kinked by the MAL. In addition to the ACACS, both the superior mesenteric artery (SMA) and the aorta were simultaneously stenosed (Figures 2A, 2C) [4,7,15-17].

We speculate that temporary impairment of CA blood flow due to thoracolumbar kyphosis correction may contribute to various transit organ dysfunction, including hyperamylasemia, POI, and liver damage, through a mechanism such as that for ACACS (Figures 2A, 2B). That thoracolumbar kyphosis correction in ACACS causes impaired blood flow in the CA and that ACACS can cause various organ damage, such as the liver, pancreas, and ileus, supports our speculation.

We propose to call the condition of transient organ dysfunction caused by thoracolumbar kyphosis correction “potential” ACACS (Figure 2B). By contrast, if there is stenosis of the aorta and SMA in addition to the CA, known as the “triple compression sign,” organ ischemia becomes irreversible and causes severe “conventional” ACACS (Figure 2C) [4]. In patients with “potential” ACACS, because the CA has rich collateral blood flow from the SMA (pancreaticoduodenal arcades), organ ischemia may be transient if SMA secures blood flow. Therefore, this compression of the CA may not necessarily lead to severe complications as it does in “conventional” ACACS (Figure 2B) [18]. We believe this is the most important difference between “potential” and “conventional” ACACS.

The reason for the elevation of the hepatobiliary enzymes may be multifactorial [7], including the correction of thoracolumbar kyphosis. That is, an intraoperative direct or indirect force injury, preexisting stenosis of CA and SMA, hypotonia, or hypovolemia may be involved. During the surgical correction, if stenosis of the CA was caused by the mechanism we proposed, hypovolemia and hypotension caused by blood loss may accelerate liver hypoperfusion. In addition, we found that operative time was significantly associated with estimated blood loss and fusion segment of the spine with Spearman's rank-order correlation (operative time - estimate blood loss: rs 0.58, p<0.01; operative time - fusion segment: rs 0.51, p<0.01). More substantial correction required a more extended fusion segment and longer operative time, leading to more blood loss and hypotension, which may cause more severe ischemia than occurs in shorter operations. However, the presence of MAL overlap, a risk factor for CA stenosis, and the fact that only thoracolumbar alignment in the sagittal plane was extracted as a significant factor for elevated LDH among multiple spinal alignments also supported the validity of our hypothesis.

As society ages, surgery for ASD will be performed more widely, and new advanced technology and established surgical technique can contribute to improving spinal correction in patients affected by ASD. Surgeons should recognize the pathophysiology of organ ischemia because its progression can lead to severe “conventional” ACACS. “Potential” ACACS is less clinically important but can sometimes lead to “conventional” ACACS, resulting in death [4]. Care should be taken not to overcorrect the thoracolumbar spine in the sagittal plane. Contrast-enhanced CT was the most effective way to detect compression of major arteries like the CA and SMA [4,7,15,17]. When abnormal laboratory values such as high LDH or atypical abdominal symptoms persist after spinal surgery, “potential” or “conventional” ACACS should be suspected, and contrast-enhanced CT is recommended.

There are some limitations to our study. Firstly, the study design was retrospective and single-center, meaning that it cannot demonstrate causality and the findings may not be applicable to other institutions. The retrospective nature of the study resulted in a lot of data loss within one or two days post-surgery. Consequently, for analytical purposes, we were compelled to select data one week after surgery. Secondly, we found that only changes in serum levels of LDH were associated with spinal alignment. We found that LDH could be elevated in ischemic hepatitis, but this is less specific than AST and ALT [19]. Additionally, we had no detailed data on LDH isozyme. Third, preoperative factors, such as the location of origin of the CA and SMA, arteriosclerosis of the CA, and stenosis of the CA were not investigated [20-22]. According to previous reports, there are many anatomical variants of the CA and SMA. Those variants may prove to be preoperative risk factors for blood flow disturbance caused by spinal surgery. Fourth, in most patients in our study, there were no clinically severe symptoms recorded. Moreover, none of the patients had to delay oral feeding for a long time after surgery. Therefore, detailed investigations, such as contrast-enhanced CT, were not performed after surgery, and we were not able to confirm stenosis of the CA. Nevertheless, to our knowledge, this is the first study about the association between serum levels of hepatobiliary enzymes and thoracolumbar spinal alignment in the sagittal plane. Our present findings may provide important clues to elucidate the pathophysiology of “potential” ACACS.

## Conclusions

We demonstrated that changes in hepatobiliary enzymes after corrective deformity surgery for ASD, especially changes in LDH, were associated with changes in TLK and preoperative T12-L1 kyphosis. Greater sagittal correction of TLK and larger preoperative T12-L1 kyphosis were factors responsible for the elevation of LDH serum levels. Additionally, MAL overlap had a relationship with the elevation of LDH serum levels, which may point to transient organ ischemia. Spine surgeons should understand that manipulations of sagittal correction for ASD can affect the location of major arteries, such as the CA and SMA, and maybe the reason for multiple organic complications. Although few patients had clinically severe symptoms, “potential” ACACS due to a temporary blood flow disturbance can occur. If abnormal laboratory values such as high LDH or atypical abdominal symptoms persist following spinal surgery, “potential” or “conventional” ACACS should be suspected, and contrast-enhanced CT should be performed.
